# Micronutrients Selenomethionine and Selenocysteine Modulate the Redox Status of MCF-7 Breast Cancer Cells

**DOI:** 10.3390/nu12030865

**Published:** 2020-03-24

**Authors:** Daniel Gabriel Pons, Carmen Moran, Marina Alorda-Clara, Jordi Oliver, Pilar Roca, Jorge Sastre-Serra

**Affiliations:** 1 Grupo Multidisciplinar de Oncología Traslacional, Institut Universitari d’Investigació en Ciències de la Salut (IUNICS), Universitat de les Illes Balears, E-07122 Palma de Mallorca, Illes Balears, Spain; d.pons@uib.es (D.G.P.); carmen.moran.04@gmail.com (C.M.); marina.alorda@uib.es (M.A.-C.); jordi.oliver@uib.es (J.O.); jorge.sastre@uib.es (J.S.-S.); 2 Instituto de Investigación Sanitaria de las Islas Baleares (IdISBa), Hospital Universitario Son Espases, edificio S, E-07120 Palma de Mallorca, Illes Balears, Spain; 3 Ciber Fisiopatología Obesidad y Nutrición (CB06/03) Instituto Salud Carlos III, E-28029 Madrid, Spain

**Keywords:** selenomethionine, selenocysteine, oxidative stress, antioxidant enzymes, ucp2, oxidative damage

## Abstract

Selenium is a micronutrient which is found in many foods, with redox status modulation activity. Our aim was to evaluate the effects of two chemical forms of selenoamino acids, Seleno-L-methionine and Seleno-L-cystine (a diselenide derived from selenocysteine), at different concentrations on cell viability, hydrogen peroxide production, antioxidant enzymes, UCP2 protein expression, as well as lipid and protein oxidative damage in MCF-7 breast cancer cells. Results showed that Seleno-L-methionine did not cause an increase in hydrogen peroxide production at relatively low concentrations, accompanied by a rise in the antioxidant enzymes catalase and MnSOD, and UCP2 protein expression levels. Furthermore, a decrease in protein and lipid oxidative damage was observed at 10 µM concentration. Otherwise, Seleno-L-cystine increased hydrogen peroxide production from relatively low concentrations (100 nM) to a large increase at high concentrations. Moreover, at 10 µM, Seleno-L-cystine decreased UCP2 and MnSOD protein expression. In conclusion, the chemical form of selenoamino acid and their incorporation to selenoproteins could affect the regulation of the breast cancer cell redox status. Taken together, the results obtained in this study imply that it is important to control the type of selenium-enriched nutrient consumption, taking into consideration their composition and concentration.

## 1. Introduction

Selenium is a micronutrient found in cereals, mushrooms, onion, nuts, broccoli, cabbage, garlic, fish, and meats [1]. Selenium has been associated with antioxidant, anti-inflammatory and cytostatic activity [2,3]. For all these properties ascribed to selenium, particular interest has been focused towards its role as a cancer preventive agent [1]. The antioxidant action is associated with the presence of selenium in some antioxidant enzymes that protect cells from oxidative damage [4].

Oxidative stress is a physio-pathological situation caused by an imbalance between antioxidant defenses of the cell and reactive oxygen species (ROS) production [5], and this stress is implicated in cancer development and progression [6,7,8,9]. Due to the antioxidant properties of selenium, this micronutrient could a possible candidate to serve as a chemopreventive agent against cancer, and more concretely breast cancer [10]. Seafoods and organ meats are the richest food sources of selenium, but selenium is significantly present in breads, grains, poultry, and eggs [11,12]. Soil selenium content and specification is a key factor for the incorporation of selenium in plants and, therefore, concentrations in animals is dependent on their plant intake, and therefore selenium content in plant and animal products can vary depending on the location [11,13]. The chemical form is important to establish the mechanisms by which selenium exerts its activities. Organic forms of selenium (selenomethionine and selenocysteine) are good dietary sources of selenium [14]. Most selenium is in the form of selenomethionine in animal and human tissues (skeletal muscle is the major site of selenium storage), as it can be incorporated non-specifically with the amino acid methionine in body proteins [14]. The human body is able to absorb more than 90% of selenomethionine present in food sources, as well as in multimineral supplements [15]. However, selenocysteine is reduced to generate hydrogen selenide, which is converted to selenophosphate for selenoprotein biosynthesis [16].

In animal models, Selenomethionine exerts, in general, dose-response chemoprevention effects without signs of toxicity [17], although Se-methylselenocysteine has also shown to have mammary chemoprevention [18]. Thus, selenium could have different effects over breast cancer related to initiation, promotion and progression of the disease. These effects include the inhibition of oxidative stress, the inhibition of cell proliferation and induction of apoptosis (modulating cell cycle progression, apoptotic genes and signaling molecules), modulation of immune response, and downregulation of angiogenesis-related genes [10].

Because of all of these precedents, the main objective of this study was to evaluate the effects of two chemical forms of selenoaminoacids: Seleno-L-methionine (SeMet) and Seleno-L-cystine (SeCys). The parameters studied were: cell viability, hydrogen peroxide production, antioxidant enzymes and UCP2 protein expression, and lipid and protein oxidative damage in MCF-7 breast cancer cells.

## 2. Material and Methods

### 2.1. Materials and Reagents

Seleno-L-Methionine (Se-Met) and Seleno-L-Cystine (Se-Cys) were obtained from Sigma-Aldrich (St. Louis, MO, USA). Routine chemicals were supplied by Roche (Barcelona, Spain), Panreac (Barcelona, Spain), Sigma-Aldrich and Bio-Rad Laboratories (Hercules, CA, USA). Amplex^®^ Red Hydrogen Peroxide/Peroxidase Assay Kit was purchased from Invitrogen—Molecular Probes—Thermo Fisher Scientific (Waltham, MA, USA).

### 2.2. Cell Culture and Treatments

Human breast cancer cell line MCF-7 was purchased from ATCC and maintained in Dulbecco’s modified Eagle’s medium (DMEM) with phenol red supplemented with 10% fetal bovine serum (FBS) and 1% penicillin/streptomycin cocktail in a 5% CO_2_ atmosphere at a temperature of 37 °C. All treatments were carried out when cells reached 70–80% confluence. For cell viability and ROS production assays, cells were treated with increasing concentrations of Se-Met (from 1 nM to 1 mM) or Se-Cys (from 100 pM to 50 µM). For western blot and oxidative damage analysis, cells were treated with 10 nM or 10 µM of both selenoamino acids. Vehicle-treated cells were treated with 0.001% dimethyl sufoxide (DMSO).

### 2.3. Cell Viability Assay

Cells were seeded at 15,000 cells per well in 96-well plates. The day after the seed, cells were treated with vehicle (0.001% DMSO) or increasing concentrations of each selenoamino acid for 48 h. The number of viable cells was determined by fluorescence emitted by the DNA binding dye Hoechst 33342 [19]. Briefly, after a 48 h treatment, DMEM was removed and each well was washed once with PBS. Then, 100 µl of 5 µg/mL Hoechst 33342 solution in PBS were added and incubated for 5 min at 37 °C. Fluorescence was measured at 350 nm of excitation length and 455 nm of emission length.

### 2.4. Measurement of ROS Production

Cells were seeded at 15,000 cells per well in 96-well plates. The day after the seed, cells were treated with increasing concentrations of both selenoamino acids for 48h. ROS production after the treatment was determined by Amplex^®^ Red Hydrogen Peroxide/Peroxidase Assay Kit, following manufacturer’s protocol, and described by Sastre-Serra et al. [20]. Briefly, after a 48h treatment, DMEM was removed and cells were washed with Krebs-Ringer phosphate buffer (145 mM NaCl, 4.86 mM KCl, 0.54 mM CaCl2, 1.22 mM MgSO4, 5.5 mM glucose, 5.7 mM sodium phosphate, pH 7.4). Then, 50 μM Amplex^®^ Red reagent and 0.1 U/mL HRP (horseradish peroxidase) diluted in Krebs-Ringer phosphate buffer was added to each well. The fluorescence measurement was monitored at times 0, 15, 30 and 60 min using a FLx800 microplate fluorescence reader (Bio-Tek, Winooski, VT, USA) adjusted at excitation and emission wavelengths of 570 and 585 nm, respectively, and the slope values were used for the calculations. All measurements were normalized by number of viable cells determined by the Hoechst 33342 method as described above.

### 2.5. Western Blot

Cells were seeded in 6-well plates at a density of 3.5 × 10^5^ cells/well. Then, cells were treated with vehicle (0.001% DMSO), 10 nM and 10 µM of each selenoamino acid for 48 h. After treatment, cells were harvested by scraping them with 200 μL of lysis buffer (50 mM Tris-Base pH 8.8, 1 mM EDTA-Na, 1% Igepal, 1 mM PMSF, 1 mM leupeptin, 1 mM pepstatin, 1 mM Na_3_VO_4_) on ice. The total protein content was determined using a BCA™ Protein Assay Kit (Pierce, Bonn, Germany), following manufacturer’s protocol. For Western blot analysis, 30 μg of total protein from cell lysates were solubilized in sample buffer (50% glycerol, 10% SDS, 300 mM Tris-HCl pH 6.8, 0.05% bromophenol blue, 10% of β-mercaptoethanol) and boiled for 5 min. Then, proteins were separated on a 12% SDS-PAGE and electrotransferred on nitrocellulose membranes with Trans-Blot^®^ Turbo™ Transfer System (Bio-Rad). After the electrotransfer, membranes were blocked with 5% non-fat powdered milk in Tris-buffered saline-Tween-20 (20 mM Tris–HCl, 0.13 mM NaCl and 0.05% Tween-20) (TBS-T) for 1h. The membranes were then incubated with primary antibodies (diluted in 5% BSA in TBS-T) to detect the following proteins: uncoupling protein 2 (UCP2) and tubulin as loading control (Santa Cruz Biotechnologies, CA, USA) at 1:200 and 1:1000 dilutions respectively; catalase, CuZn-superoxide dismutase (CuZnSOD) and MnSOD (Calbiochem, San Diego, CA, USA) at 1:1000 dilution. The membranes were then washed with TBS-T and incubated with rabbit or mouse secondary antibodies conjugated with horseradish peroxidase (Santa Cruz Biotechnology, Texas, CA, USA) at 1:10,000 dilution in 2% non-fat powdered milk in TBS-T. After washing membranes with TBS-T, protein bands were visualized by Immun-Star^®^ WesternC^©^ Chemiluminescent Kit Western blotting detection systems (Bio-Rad). The chemiluminescence signal was acquired with a Chemidoc XRS densitometer (Bio-Rad Laboratories) and results were analyzed with Quantity One Software (Bio-Rad).

### 2.6. Measurement of Carbonyl Groups

The presence of carbonyl groups was analyzed using an immunological method with the OxySelect^TM^ Protein Carbonyl Immunoblot kit (Cell Biolabs, San Diego, CA, USA), following the manufacturer’s protocol. Briefly, protein carbonyls of 10 µg of total protein were detected by labelling them with 2,4-dinitrophenylhydrazine (DNPH) for 5 min. Total protein was loaded and separated in a 12% SDS-PAGE and electrotransferred on a nitrocellulose membrane as described above. After de blockage of the membrane for 1 h, it was incubated with the DNP antibody (1:2000) in 5% BSA in TBS-T for 2 h and then incubated with the secondary antibody anti-mouse at 1:2000 dilution in 2% non-fat powdered milk in TBS-T for 1 h. The band detection was performed as described above in the Western blotting section.

### 2.7. Measurement of 4-Hydroxy-2Nonenal Adducts

For 4-hydroxy2-nonenal (4-HNE) adducts analysis, 30 µg of total protein were loaded in a 12% SDS-PAGE and electrotransferred as described above. After the blockage of the membrane, it was incubated with antiserum against 4-HNE (Santa Cruz Biotechnologies, CA, USA) at a dilution of 1:1000 in 5% BSA in TBS-T. Then, the membrane was incubated with the secondary antibody anti-goat at 1:10,000 dilution in 2% non-fat powdered milk in TBS-T for 1 h. The band detection was performed as describe above in the Western blotting section.

### 2.8. Statistical Analysis

The statistical analysis was performed with the Statistical Program for the Social Sciencies software for Windows (SPSS, version 24.0; SPSS Inc, Chicago, IL, USA). Data are presented as mean ± standard error of the mean (SEM). The statistical differences between vehicle- and selenoamino acids-treated cells were analyzed using a Student’s t-test. Statistical significances were set at *p* < 0.05 or at *p* < 0.1.

## 3. Results

### 3.1. Effects of Increasing Concentrations of Selenoamino Acids on Cell Viability and H2O2 Production

**Figure 1:**
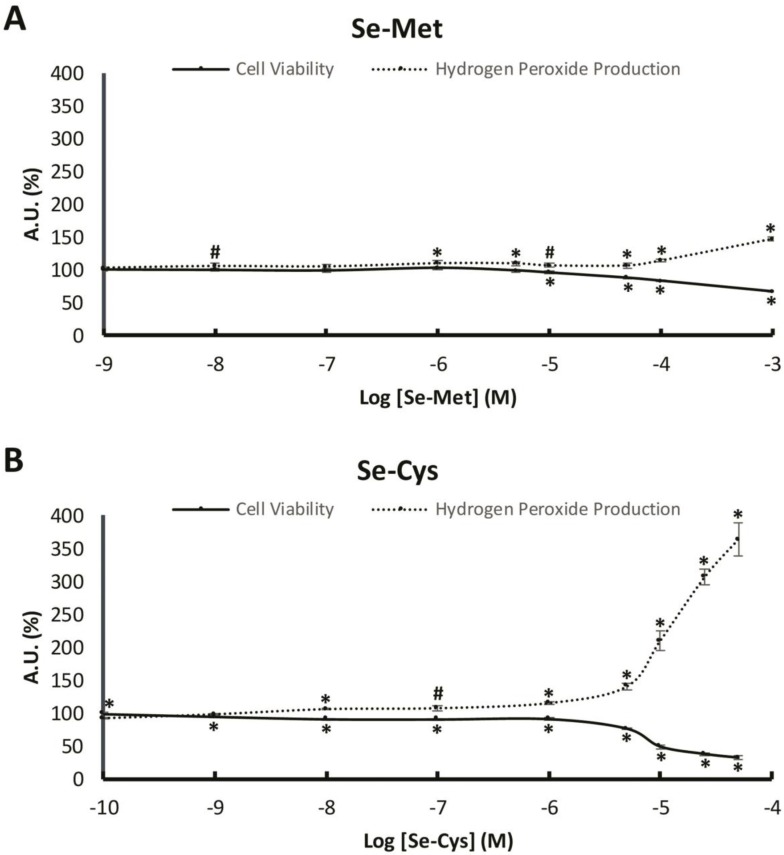
Cell viability and hydrogen peroxide production in MCF-7 breast cancer cells after selenomethionine or selenocystine treatment. (**A**) The cells were treated with vehicle or with increasing concentrations of selenomethionine (from 1 nM to 1 mM) for 48 h. (**B**) The cells were treated with vehicle (data not shown) or with increasing concentrations of selenocystine (from 100 pM to 50 µM) for 48 h. Solid line represents the cell viability and pointed line represents hydrogen peroxide production. Points in lines represent means and error bars represent SEM (*n* = 6). The value of control cells was set at 100% (data not shown) and the rest of values were calculated referenced to the control set at 100%. * Significant differences between selenoamino acid treated and control cells (Student’s t-test; *p* ≤ 0.05). # Significant differences between selenoamino acid treated and control cells (Student’s t-test; *p* ≤ 0.1). AU: Arbitrary Units.

Figure 1A shows that increasing SeMet concentrations up to a concentration of 10 µM of SeMet did not cause any significant changes in cell viability. Nonetheless, above this concentration (10 µM) of SeMet, cell viability gradually decreased until a drop of 33% in the highest SeMet concentration tested, 1 mM. Moreover, H_2_O_2_ production was not affected under low concentrations of SeMet and this parameter increased slightly in a concentration-dependent manner, until reaching a maximum increase of 47% with the highest SeMet concentration tested, 1 mM.

In Figure 1B, for the SeCys treatment, a decrease in cell viability can be observed at the concentration of 1 nM and this decrease was higher in a concentration-dependent manner, until a drop of 68% was noted in the highest SeCys concentration tested, at 50 µM. At high concentrations of SeCys, there was an increase in H_2_O_2_ production in a concentration-dependent manner, and this became increasingly more pronounced from 5 µM (+40%) to 50 µM (+264%).

### 3.2. Effects of Selenoamino Acids on Antioxidant Enzymes and UCP2 Protein Levels

**Figure 2:**
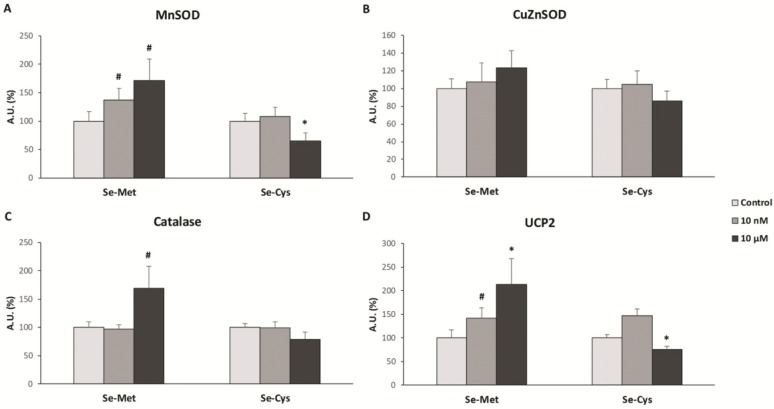
Antioxidant enzymes and UCP2 protein levels in MCF-7 breast cancer cell lines after treatment with selenoamino acids at 10 nM and 10 μM. The cells were treated with vehicle (clear grey bars), selenoamino acid at 10 nM (dark grey bars) or selenoamino acid at 10 μM (black bars) for 48 h. (**A**) Manganese Superoxide Dismutase (MnSOD). (**B**) Copper-Zinc Superoxide Dismutase (CuZnSOD). (**C**) Catalase. (**D**) Uncoupling protein 2 (UCP2). The proteins levels were measured by Western Blot. Bars represent means and error bars represent SEM (*n* = 6). The value of control cells was set at 100% and the rest of values were calculated referenced to the control set at 100%. * Significant differences between selenoamino acid treated and control cells (Student’s t-test; *p* ≤ 0.05). # Significant differences between selenoamino acid treated and control cells (Student’s t-test; *p* ≤ 0.1). AU: Arbitrary Units.

For the analysis of the antioxidant enzymes and UCP2 protein levels, we chose two concentrations of each Se-aminoacid, 10 nM and 10 µM, representative of the results obtained in the cell viability and H_2_O_2_ production analysis. Figure 2 displays the results obtained in the protein levels of manganese superoxide dismutase (MnSOD), copper-zinc superoxide dismutase (CuZnSOD), catalase and uncoupling protein 2 (UCP2) by western blot analysis.

**Figure 3:**
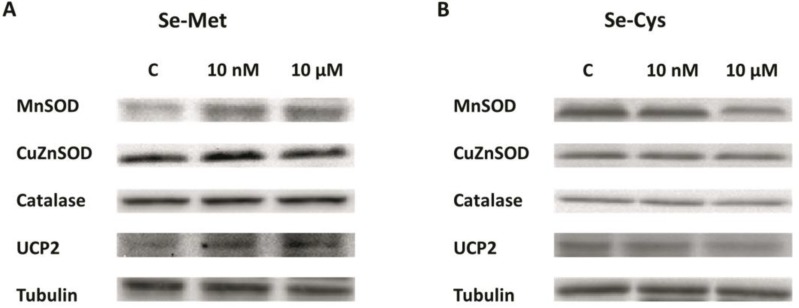
Western blot cropped representative bands of (**A**) selenomethione-treated and (**B**) selenocystine-treated MCF-7 breast cancer cell line are shown.

Figure 2A represents the MnSOD protein expression levels, showing an increase in SeMet-treated cells at both concentrations (+37% 10 nM and +71% 10 µM) and a decrease in 10 µM SeCys-treated cells (−34%). In Figure 2B the CuZnSOD protein levels are displayed which did not undergo any significant change for either SeMet or SeCys treatment in MCF-7 cells. In Figure 2C the catalase protein levels are presented, indicating only one, albeit a very noteworthy, statistically significant change in catalase protein level, with a 10 µM SeMet treatment (+69%). Figure 2D shows a remarkably significant increase in UCP2 protein expression levels when cells were treated with SeMet at both concentrations (+42% for 10 nM and +113% for 10 µM), in addition to a decrease in UCP2 protein levels when cells were treated with SeCys at 10 µM (−24%). Western blot cropped representative bands of selenomethione-treated and selenocystine-treated MCF-7 breast cancer cells can be observed in Figure 3.

### 3.3. Effects of Selenoamino Acids Treatment on Protein and Lipid Oxidative Damage

**Figure 4:**
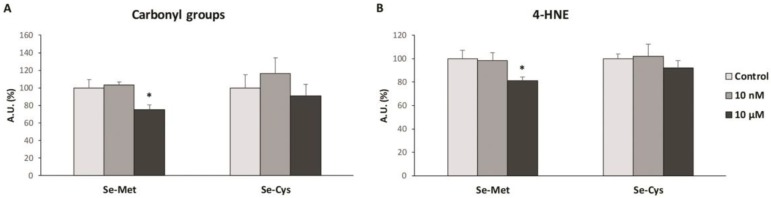
Protein and lipid oxidative damage in MCF-7 breast cancer cell lines after treatment with selenoamino acids at 10 nM and 10 μM. The cells were treated with vehicle (clear grey bars), selenoamino acid at 10 nM (dark grey bars) or selenoamino acid at 10 μM (black bars) for 48h. (**A**) Protein carbonyl groups formation. (**B**) 4-Hydroxy-2Nonenal adducts formation (4-HNE). Bars represent means and error bars represent SEM (*n* = 6). The value of control cells was set at 100% and the rest of values were calculated referenced to the control set at 100%. * Significant differences between selenoamino acid treated and control cells (Student’s t-test; *p* ≤ 0.05). AU: Arbitrary Units.

As the protein expression levels change with selenoamino acid treatment, for the analysis of the oxidative damage, we chose the same two concentrations of each selenoamino acid, 10 nM and 10 µM, in order to obtain comparable results. In Figure 4 the protein and lipid oxidative damage is represented, analyzed with protein carbonyls and 4-HNE formation respectively.

Figure 4A displays the protein carbonyls formation, showing that after SeMet treatment at 10 µM, MCF-7 cells presented a lower carbonyls formation (−25%), while there were no significant changes after SeCys treatment, for either at the 10 nM or at 10 µM concentrations. In Figure 4B, the 4-HNE formation are presented, indicating a decrease after treatment with SeMet at 10 µM (−19%), with no significant differences after SeCys treatment at both concentrations studied.

## 4. Discussion

The results obtained in this study have revealed different biological effects of selenium depending on the chemical form of the selenoamino acid. Thus, seleno-L-methionine (SeMet) did not modify the cell viability and hydrogen peroxide production, except at the highest concentrations, in MCF-7 breast cancer cells. Moreover, SeMet treatment at 10 µM produced a decrease in oxidative damage in lipids and proteins, which was accompanied by an increase of the UCP2 and antioxidant enzymes protein expression. However, seleno-L-cystine (SeCys), a diselenide derived from selenocysteine, produced a decrease in cell viability and an increase in hydrogen peroxide production at a concentration of 1 nM and above in a concentration-dependent manner. In this case, SeCys treatment at 10 µM did not produce any statistically significant change in the oxidative damage of both lipids and proteins, although it did decrease the UCP2 and antioxidant enzymes protein expression.

In our study, we found that SeMet did not significantly increase hydrogen peroxide production, except at very high concentrations (>100 µM). The observed effects of SeMet are similar to those found twenty years ago by Stewart et al. [21], who determined that SeMet neither resulted in cell death nor DNA oxidative damage in BALB/c MK-2 cells (mouse keratinocyte cell line), compared to other selenium compounds such as selenite and selenocystamine [21]. This is plausible, as SeMet is a non-catalytic compound and does not generate superoxide anion [22]. Moreover, we found an overexpression of some antioxidant enzymes when MCF-7 cells were treated with SeMet, especially at 10 µM, which suggests that this selenoamino acid could, in some way, improve the redox state of these cells through the overexpression or the extension of the half-life of these antioxidant enzymes. These results are in concordance with a recent study carried out in chicken myocardial tissue that suggests that SeMet reduces hydrogen peroxide production and lipid oxidative damage, in addition to an increase in the enzymatic activity of antioxidant enzymes such as SOD and catalase [23]. Our results demonstrate that, in addition to an increase in the protein levels of antioxidant enzymes and UCP2 with SeMet treatment, concomitant oxidative damage in macromolecules such as proteins and lipids also decrease, which corroborates the antioxidant properties of this molecule observed in older and recent studies [23,24].

Nonetheless, the other selenoamino acid studied, selenocysteine, presented very different effects compared to SeMet. It is important to note that cells reduce selenocystine to selenocysteine through reactions with glutathione and cysteine, so that selenocysteine form exerts the main effects over the cells [25]. SeCys treatment caused an increase in hydrogen peroxide production at relatively low concentrations (from 1 µM), which became greatly evident at higher concentrations. Moreover, cell viability was compromised. In a previous study carried out in MCF-7 cell-based breast cancer xenograft model in nude mice, methylselenocysteine led to synergistic inhibition of tumor growth that was associated with inhibition of cyclin D1 as well as inhibition of cell proliferation and induction of apoptosis [26]. Both results, i.e., the increase in hydrogen peroxide production and the decrease in cell viability after SeCys treatment, suggest that SeCys could diminish cancer cell proliferation through the raise in oxidative stress, although SeCys is known to form part of some antioxidant enzymes [27]. Other studies have proven the effects of SeCys in the rise in ROS production measured with DCFH-DA, a generalist dye detecting ROS levels, in MCF-7 and other cancer cell types [28]. In addition to all these evidences, Chen and Wong found that selenocystine treatment increased MCF-7 breast cancer cells apoptosis by modulation of ERK and Akt phosphorylation or with the involvement of p53 phosphorylation and ROS generation [29,30], supporting the results obtained in this study.

The different effects observed in both selenoamino acids could be due to the capacity of SeCys to form hydrogen selenide and, from this molecule, different prooxidant products can be created, producing reactive oxygen species such as superoxide anion, among others [31]. In contrast, SeMet would be mostly incorporated into antioxidant proteins in a non-specific way, and higher concentrations of this selenium compound would be required to produce ROS since this selenoamino acid would first need to be converted to SeCys, with selenium metabolism being one of the key factors to understand the role of selenoamino acids in human health and cancer chemoprevention [32]. In this context, other authors have demonstrated that sodium selenite at high nanomolar to low micromolar concentrations down-regulate the expression of MnSOD and UCP2 proteins [33]. According to the data obtained, and taking in consideration other previously published studies, selenomethionine could be considered, in the future, as a possible food supplement for the improvement of the redox activity of cells. On the other hand, selenocystine could be considered as an adjuvant treatment against breast tumors through a dramatic increase in ROS production in cancer cells and, therefore, induction of the cell death ways.

The present study has some limitations, as it has been done in in vitro conditions. To investigate the real effects of these selenoamino acids, in vivo studies are necessary and the research in the expression of some enzymes such as the methionine gamma-lyase, which modifies the chemical structure and therefore the biological function of these selenoamino acids [34]. In fact, a treatment based in the combination of selenomethione with methionine gamma-lyase inhibited the tumor growth in rodents and prolonged their survival [35]. Moreover, another enzyme found in the liver, cystathione gamma-lyase, could play a crucial role in the effects of these selenoamino acids [36]. This enzyme catalyzes the transformation of the SeMet in SeCys, so its expression could be important in the final effects of both selenoamino acids [36].

## 5. Conclusions

In conclusion, the micronutrients selenomethionine and selenocysteine could play a crucial role in the regulation of the redox status in breast cancer cells. Selenomethionine increases the antioxidant enzymes and UCP2 protein expression, reducing the oxidative damage in both lipids and proteins, suggesting that this molecule could improve the redox status of cells. On the other hand, selenocystine treatment causes a drastic increase in ROS production followed by a dramatic drop in breast cancer cells viability at relative high concentrations. Moreover, selenocystine decrease the antioxidant enzymes and UCP2 protein expression, suggesting that this selenoamino acid could increase the oxidative stress in cells. Taken together, the results obtained in this study imply that it is important to control the type of selenium-enriched nutrient consumption, taking into consideration their composition and concentration.
